# Supplementary material for: Human Cadaveric Donor Cornea Derived Extra Cellular Matrix Microparticles for Minimally Invasive Healing/Regeneration of Corneal Wounds
Source: Biomolecules. 2021 Apr 2;11(4):532. doi: 10.3390/biom11040532 (PMC8066719; doi:10.3390/biom11040532)
Supplement: Supplementary file 1 [file biomolecules-11-00532-s001.pdf]

Supplementary data

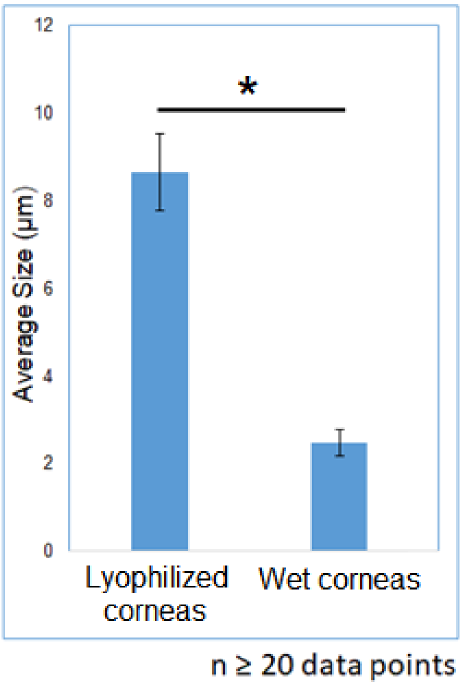

Figure S1. Average size of freeze milled corneal dECM microparticles obtained from lyophilized corneas and wet corneas as measured through scanning electron microscopy. Data are represented as mean ± SE of 20 different particles/group. **p ≤ 0.05 denotes significant differences observed between lyophilized and wet corneas with p value 0.02.

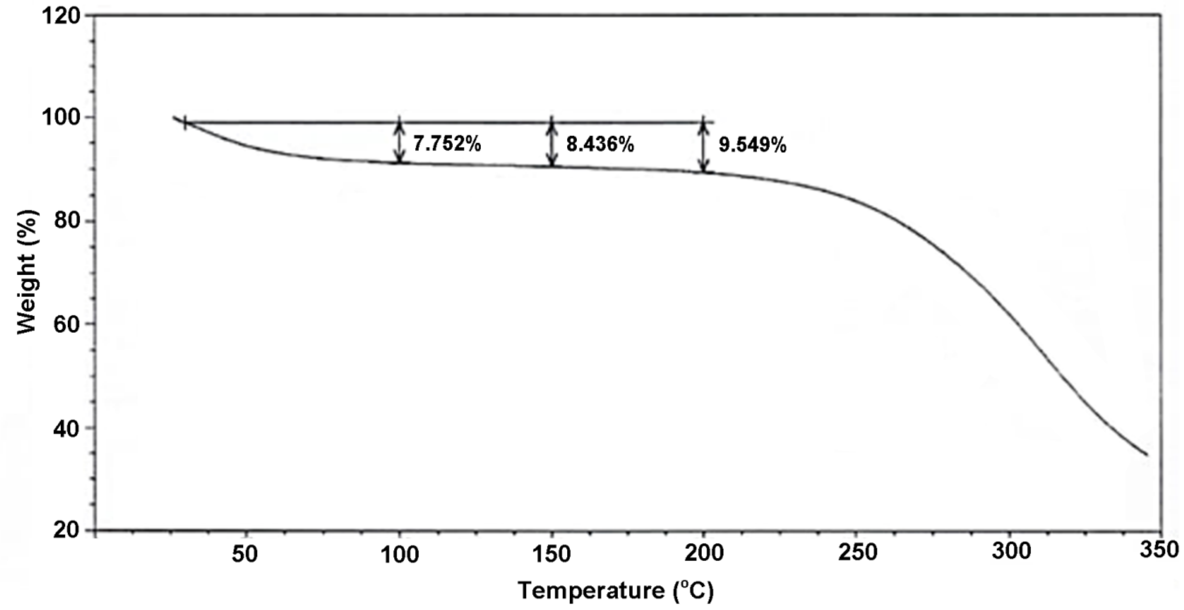

Figure S2. Thermogravimetric analysis on physically milled dECM microparticles depicting percentage moisture content at different temperatures.

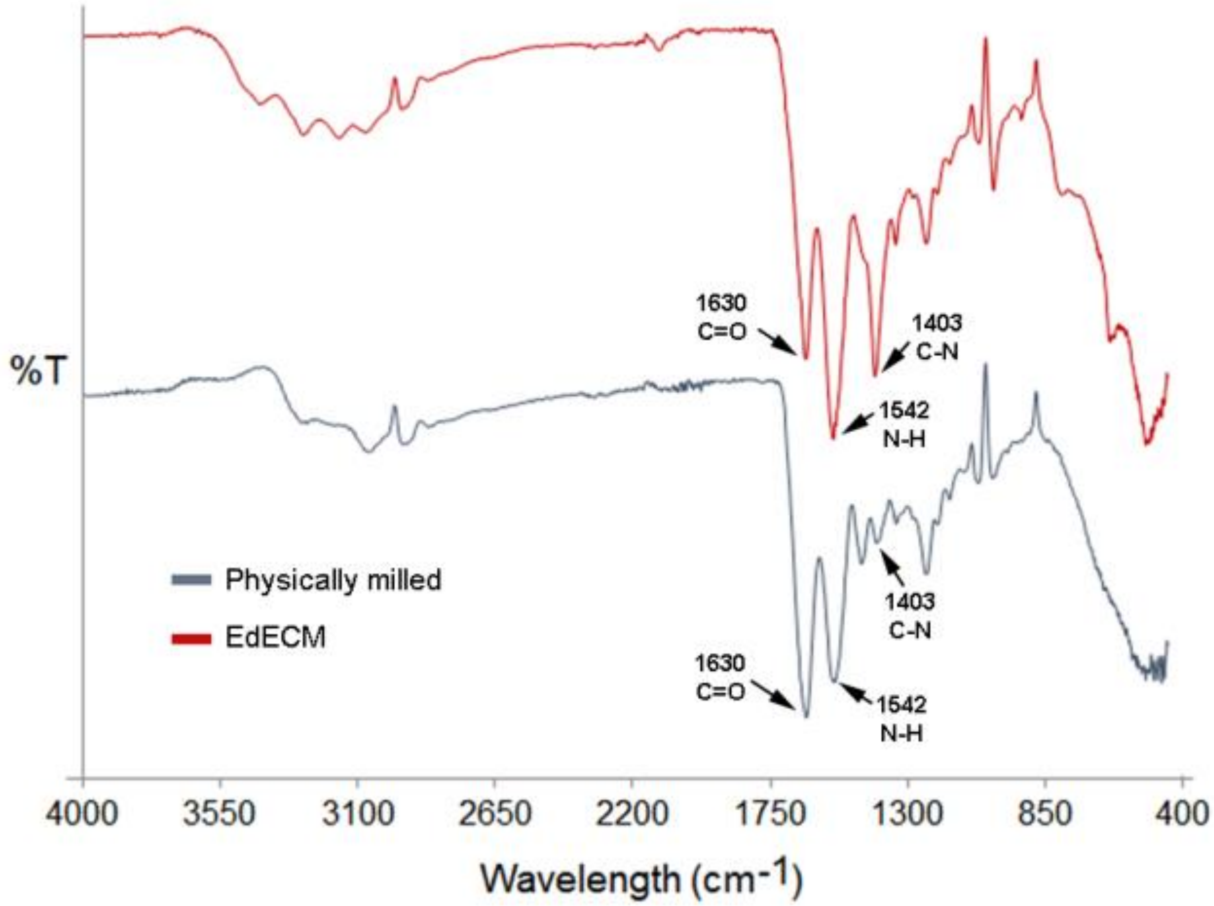

Figure S3. FTIR spectroscopy of physically milled and enzymatically digested dECM microparticles. FTIR spectra depicted characteristic C=O stretching vibration of the amide group at 1630 cm^{-1}, while the out-of-phase and in-phase combination of N-H bending and C-N stretching vibration were observed at 1542 cm^{-1} and 1403 cm^{-1} in both groups, respectively.

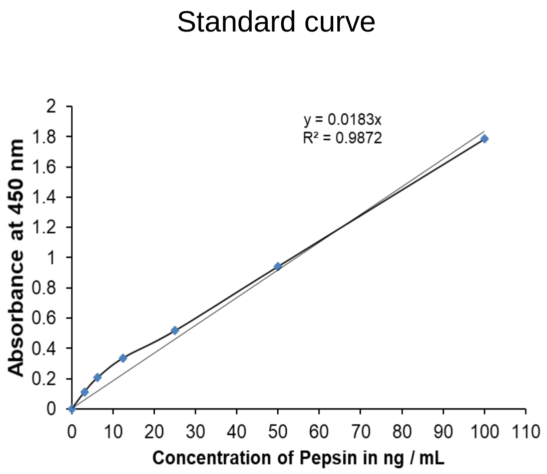

Std (ng/mL)/sample	Mean - Blank mean	Conc. of Pepsin in ng/mL
100	1.7835	
50	0.9415	
25	0.5165	
12.5	0.3375	
6.25	0.2065	
3.125	0.1135	
0	0	
EdECM	0.6	32.787
NC - 100 ng/mL	0.042	2.295

Figure S4. Quantification of porcine pepsin using sandwich ELISA. Amount of pepsin detected in 30 mg EdECM = 32.787 ng/mL, which is equivalent to 0.0001 %.

Table S1: Components of human cornea dECM after pepsin digestion via mass spectrometry analysis

1. Keratocan	25. Protein cdv3 homolog isoform a	50. Dermatopontin
2. Collagen alpha-2(V) chain	26. Annexin A5	51. Fibromodulin
3. Collagen alpha-1(I) chain	27. Guanine nucleotide-binding protein-like 3-like protein	52. Ferritin heavy chain
4. Collagen alpha-2(I) chain	28. Actin, cytoplasmic 2	53. Lumican
5. Collagen alpha-1(III) chain	29. Pyruvate kinase PKM isoform d	54. Prolargin
6. Transforming growth factor-beta-induced protein ig-h3	30. Gamma-secretase subunit aph-1a isoform 4	55. Trypsin-1
7. Keratin, type I cytoskeletal 9	31. Complement C4-A isoform 2	56. Serine protease HTRA1
8. Complement C3	32. Carbonic anhydrase 1 isoform c	57. C-type lectin domain family 11 member A
9. Ferritin light chain	33. Carbonic anhydrase 2 isoform 2	58. Extracellular superoxide dismutase [cu-zn]
10. Transthyretin	34. Calpain small subunit 1 isoform 1	58. Thrombospondin-1
11. Keratin, type I cytoskeletal 10	35. Alpha-enolase isoform 1	59. Ubiquitin-like modifier-activating enzyme 1
12. Keratin, type II cytoskeletal 2 epidermal	36. Neuroblast differentiation-associated protein AHNAK isoform 1	60. Vimentin
13. Serum albumin	37. Protein ambp	61. Collagen alpha-1(xii) chain long isoform
14. Hemoglobin subunit beta	38. Serum amyloid p-component	62. Mediator of rna polymerase ii transcription subunit 12
15. Hemoglobin subunit delta	40. Apolipoprotein d	63. Basement membrane-specific heparan sulfate proteoglycan core protein isoform b
16. Hemoglobin subunit alpha	41. ADP-ribosylation factor 5	64. Peroxiredoxin-2
17. Aldehyde dehydrogenase, dimeric NADP-preferring	42. ATP synthase subunit beta, mitochondrial	65. Keratin, type II cytoskeletal 1
18. Annexin A1	43. Biglycan	66. Sushi repeat-containing protein SRPX2
19. Prostaglandin-H2 D-isomerase	44. Complement component C9	67. Procollagen galactosyltransferase 1
20. Glucosidase 2 subunit beta isoform 2	45. Catalase	68. Mimecan isoform 2
21. Annexin A2 isoform 2	46. Clusterin	69. Collagen alpha-1(II) chain isoform 2
22. Serotransferrin	47. Collagen alpha-1(vi) chain	70. Collagen alpha-3(vi) chain isoform 5
23. Alpha-1-antichymotrypsin	48. Collagen alpha-2(vi) chain isoform 2c2	71. Mam domain-containing protein 2
24. Alpha-1-antitrypsin	49. Decorin isoform a	72. Olfactomedin-like protein 1

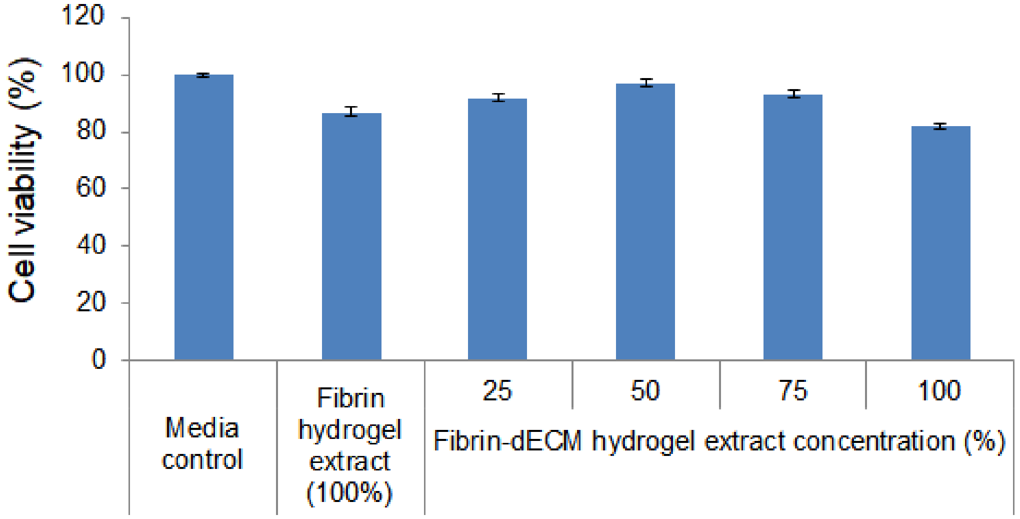

Figure S5. Cytotoxicity potential of fibrin and fibrin-dECM hydrogel extracts and their degradation products as evaluated by MTT assay on L929 cell cultures at four different concentrations. Data are represented as means ± SE with n = 6 samples/group.

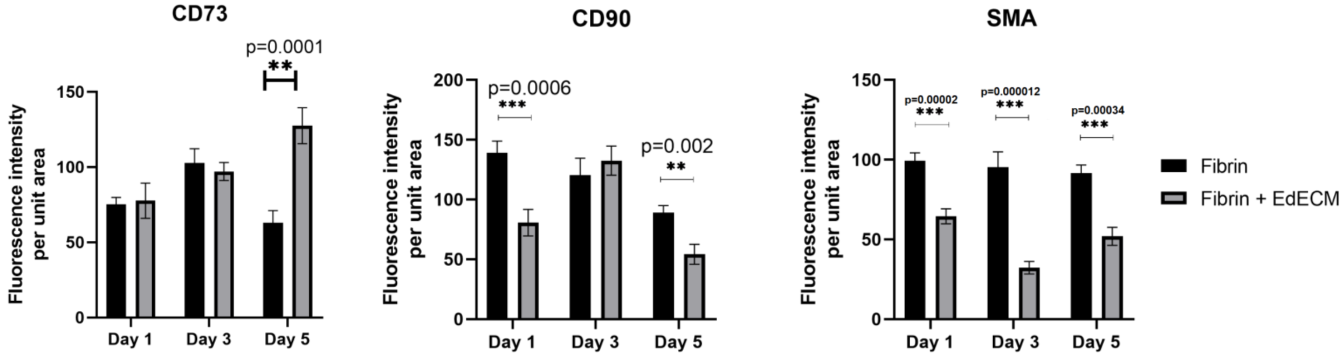

Figure S6. Quantification of immunofluorescence signals from CD73, CD90 and α-SMA from hCSC encapsulated fibrin and fibrin+EdECM hydrogel samples using ImageJ. Data are represented as means ± SE with n ≥ 15 regions of interest format least three different images. *p ≤ 0.05 denotes significant differences observed between fibrin and fibrin-EdECM hydrogels.

Tables S2: Bacterial reverse mutation test study report

Summary of colony counts of revertants (polar extraction): Plate Incorporation Method

Treatment	Test Concentration (%/plate)/Volume		No. of Revertants (Mean of 3 Plates)									
			**With S9**					**Without S9**				
			Salmonella typhimurium				***E.coli* WP2 uvrA (pKM 101)**	***Salmonella typhimurium***				***E.coli* WP2 uvrA (pKM 101)**
			**TA 98**	**TA 100**	**TA 1535**	**TA 1537**		**TA 98**	**TA 100**	**TA 1535**	**TA 1537**	
**Solvent Control**	**100 µL of Normal Saline**	**Mean**	25.7	110.7	22.0	9.3	171.7	24.0	104.3	22.3	11.0	157.0
		**±SD**	4.0	3.8	2.6	1.2	5.7	2.6	4.7	1.2	1.0	5.6
	**Lawn Intensity**		4+	4+	4+	4+	4+	4+	4+	4+	4+	4+
**Fibrin-dECM Hydrogel**	**100 µL of Polar Extract**	**Mean**	21.7	99.7	21.7	7.3	176.7	20.3	96.7	22.0	5.3	172.7
		**±SD**	1.5	9.9	2.9	0.6	2.5	0.6	3.5	2.0	0.6	3.1
	**Fold Increase**		0.8	0.9	1.0	0.8	1.0	0.8	0.9	1.0	0.5	1.1
	**Lawn Intensity**		4+	4+	4+	4+	4+	4+	4+	4+	4+	4+
**Positive Control**	**100 µL of Respective Positive Control**	**Mean**	375.7	404.7	130.7	116.0	408.7	362.7	397.3	116.7	103.0	401.7
		**±SD**	4.0	10.3	5.5	7.0	7.5	8.0	7.1	4.2	5.6	9.7
	**Fold Increase**		14.6	3.7	5.9	12.4	2.4	15.1	3.8	5.2	9.4	2.6
	**Lawn Intensity**		4+	4+	4+	4+	4+	4+	4+	4+	4+	4+

Values of Revertants are in Mean±SD

Positive controls:
For with S9:
For *Salmonella typhimurium* TA98, TA100, TA1535 and TA1537 = 4 µg/plate of 2 Aminoanthracene
For *Escherichia coli* WP2 uvrA (pKM101) strain +S9= 30 µg/plate of 2 Aminoanthracene
For without S9:
For TA98: 2 µg/plate of 2 Nitrofluorene
For TA100 and TA1535: 1µg/plate of Sodium azide.
For TA1537: 50 µg/plate of 9 Aminoacridine
For *Escherichia coli* WP2 uvrA (pKM101): 5 µg/plate of 4-Nitroquinoline N-oxide
Lawn intensity: 4+ = Thick lawn: Distinguished by a healthy (Normal) background lawn comparable to solvent control plates.

Summary of colony counts of revertants (non-polar extraction): Plate Incorporation Method

Treatment	Test Concentration (%/plate)/Volume		No. of Revertants (Mean of 3 Plates)									
			With S9					Without S9				
			Salmonella typhimurium				*E.coli* WP2 uvrA (pKM 101)	*Salmonella typhimurium*				*E.coli* WP2 uvrA (pKM 101)
			TA 98	TA 100	TA 1535	TA 1537		TA 98	TA 100	TA 1535	TA 1537	
Solvent Control	100 µL of Sesame Oil	Mean	27.0	110.0	21.3	9.0	174.7	24.0	102.7	21.3	9.3	163.0
		±SD	2.6	8.0	4.2	1.0	4.5	1.7	5.0	3.5	2.3	5.6
	Lawn Intensity		4+	4+	4+	4+	4+	4+	4+	4+	4+	4+
Fibrin-dECM Hydrogel	100 µL of Non-polar Extract	Mean	26.0	113.3	23.3	8.3	171.3	24.7	111.7	18.7	8.7	166.7
		±SD	1.0	4.7	3.8	2.5	4.2	1.2	4.6	0.6	2.1	7.6
	Fold Increase		1.0	1.0	1.1	0.9	1.0	1.0	1.1	0.9	0.9	1.0
	Lawn Intensity		4+	4+	4+	4+	4+	4+	4+	4+	4+	4+
Positive Control	100 µL of Respective Positive Control	Mean	375.7	404.7	130.7	116.0	408.7	362.7	397.3	116.7	103.0	401.7
		±SD	4.0	10.3	5.5	7.0	7.5	8.0	7.1	4.2	5.6	9.7
	Fold Increase		13.9	3.7	6.1	12.9	2.3	15.1	3.9	5.5	11.0	2.5
	Lawn Intensity		4+	4+	4+	4+	4+	4+	4+	4+	4+	4+

Values of Revertants are in Mean±SD

Positive controls:
For with S9:
For *Salmonella typhimurium* TA98, TA100, TA1535 and TA1537 = 4 µg/plate of 2 Aminoanthracene
For *Escherichia coli* WP2 uvrA (pKM101) strain +S9= 30 µg/plate of 2 Aminoanthracene
For without S9:
For TA98: 2 µg/plate of 2 Nitrofluorene
For TA100 and TA1535: 1µg/plate of Sodium azide.
For TA1537: 50 µg/plate of 9 Aminoacridine
For *Escherichia coli* WP2 uvrA (pKM101): 5 µg/plate of 4-Nitroquinoline N-oxide
Note: Positive control values are from the polar extaction treatment and are included in the non-polar extraction treatment for comparison with the treatment groups for fold increase.

Lawn intensity: 4+ = Thick lawn: Distinguished by a healthy (Normal) background lawn comparable to solvent control plates.

Summary of colony counts of revertants (polar extraction): Preincubation method

Treatment	Test Concentration (%/plate)/Volume		No. of Revertants (Mean of 3 Plates)									
			With S9					Without S9				
			Salmonella typhimurium				***E.coli* WP2 uvrA (pKM 101)**	*Salmonella typhimurium*				***E.coli* WP2 uvrA (pKM 101)**
			**TA 98**	**TA 100**	**TA 1535**	**TA 1537**		**TA 98**	**TA 100**	**TA 1535**	**TA 1537**	
**Solvent Control**	**100 µL of Normal Saline**	**Mean**	22.0	111.0	18.3	7.3	171.0	18.7	108.7	19.0	6.7	161.7
		**±SD**	2.6	6.0	1.2	0.6	3.0	0.6	3.1	2.6	1.2	6.0
	**Lawn Intensity**		4+	4+	4+	4+	4+	4+	4+	4+	4+	4+
**Fibrin-dECM Hydrogel**	**100 µL of Polar Extract**	**Mean**	22.7	107.0	19.3	7.3	170.0	19.0	102.0	20.3	5.7	158.3
		**±SD**	2.1	4.4	1.2	1.5	5.6	1.0	3.0	0.6	0.6	2.1
	**Fold Increase**		1.0	1.0	1.1	1.0	1.0	1.0	0.9	1.1	0.9	1.0
	**Lawn Intensity**		4+	4+	4+	4+	4+	4+	4+	4+	4+	4+
**Positive Control**	**100 µL of Respective Positive Control**	**Mean**	373.0	405.7	130.7	114.0	402.7	361.7	401.0	120.7	106.7	397.3
		**±SD**	10.8	4.0	6.7	5.6	7.0	6.7	6.6	7.0	5.9	7.5
	**Fold Increase**		17.0	3.7	7.1	15.5	2.4	19.4	3.7	6.4	16.0	2.5
	**Lawn Intensity**		4+	4+	4+	4+	4+	4+	4+	4+	4+	4+

Values of Revertants are in Mean±SD

Positive controls:
For with S9:
For *Salmonella typhimurium* TA98, TA100, TA1535 and TA1537 = 4 µg/plate of 2 Aminoanthracene
For *Escherichia coli* WP2 uvrA (pKM101) strain +S9= 30 µg/plate of 2 Aminoanthracene
For without S9:
For TA98: 2 µg/plate of 2 Nitrofluorene
For TA100 and TA1535: 1 µg/plate of Sodium azide.
For TA1537: 50µg/plate of 9 Aminoacridine
For *Escherichia coli* WP2 uvrA (pKM101): 5 µg/plate of 4-Nitroquinoline N-oxide

Lawn intensity: 4+ = Thick lawn: Distinguished by a healthy (Normal) background lawn comparable to solvent control plates.

Summary of colony counts of revertants (non-polar extraction): Preincubation method

Treatment	Test Concentration (%/plate)/Volume		No. of Revertants (Mean of 3 Plates)									
			With S9					Without S9				
			Salmonella typhimurium				*E.coli* WP2 uvrA (pKM 101)	*Salmonella typhimurium*				*E.coli* WP2 uvrA (pKM 101)
			TA 98	TA 100	TA 1535	TA 1537		TA 98	TA 100	TA 1535	TA 1537	
**Solvent Control**	**100 µL of Sesame Oil**	**Mean**	23.7	110.3	22.7	7.3	159.7	21.3	104.3	20.7	5.3	163.0
		**±SD**	1.2	3.2	2.5	1.2	8.0	2.5	4.9	1.5	0.6	6.6
	**Lawn Intensity**		4+	4+	4+	4+	4+	4+	4+	4+	4+	4+
**Fibrin-dECM Hydrogel**	**100 µL of Non-polar Extract**	**Mean**	22.3	106.3	19.7	8.3	160.7	20.3	102.7	19.7	7.0	158.0
		**±SD**	2.5	6.0	2.1	1.2	7.0	1.2	3.1	1.2	1.0	2.0
	**Fold Increase**		0.9	1.0	0.9	1.1	1.0	1.0	1.0	1.0	1.3	1.0
	**Lawn Intensity**		4+	4+	4+	4+	4+	4+	4+	4+	4+	4+
**Positive Control**	**100 µL of Respective Positive Control**	**Mean**	373.0	405.7	130.7	114.0	402.7	361.7	401.0	120.7	106.7	397.3
		**±SD**	10.8	4.0	6.7	5.6	7.0	6.7	6.6	7.0	5.9	7.5
	**Fold Increase**		15.8	3.7	5.8	15.5	2.5	17.0	3.8	5.8	20.0	2.4
	**Lawn Intensity**		4+	4+	4+	4+	4+	4+	4+	4+	4+	4+

Values of Revertants are in Mean±SD
Positive controls:
For with S9:
For *Salmonella typhimurium* TA98, TA100, TA1535 and TA1537 = 4 µg/plate of 2 Aminoanthracene
For *Escherichia coli* WP2 uvrA (pKM101) strain +S9= 30 µg/plate of 2 Aminoanthracene
For without S9:
For TA98: 2 µg/plate of 2 Nitrofluorene
For TA100 and TA1535: 1µg/plate of Sodium azide.
For TA1537: 50 µg/plate of 9 Aminoacridine
For *Escherichia coli* WP2 uvrA (pKM101): 5 µg/plate of 4-Nitroquinoline N-oxide

Lawn intensity: 4+ = Thick lawn: Distinguished by a healthy (Normal) background lawn comparable to solvent control plates.

Note: Positive control values are from the polar extaction treatment and are included in the non polar extraction treatment for comparison with the treatment groups for fold increase.

Table S3. Skin sensitization test study report

SKIN REACTIONS SCORING RECORD

Group, Sex & Treatment	Animal No.	Site	Intra-dermal Induction (Day 1)				Topical Induction (Day 8)				Challenge (Day 22)			
			24 (± 2 hrs)		48 (± 2 hrs)		1 hr		24 hrs		24 (± 2 hrs)		48 (± 2 hrs)	
			Ery	Ede	Ery	Ede	Ery	Ede	Ery	Ede	RF ant	RF post	RF ant	RF post
G1, Female & Polar Solvent Control	Gb9753	1	1	1	1	1								
		2	0	0	0	0	0	0	0	0	0	0	0	0
		3	1	1	1	1								
	Gb9754	1	1	1	1	1								
		2	0	0	0	0	0	0	0	0	0	0	0	0
		3	1	1	1	1								
	Gb9755	1	1	1	1	1								
		2	0	0	0	0	0	0	0	0	0	0	0	0
		3	1	1	1	1								
	Gb9756	1	1	1	1	1								
		2	0	0	0	0	0	0	0	0	0	0	0	0
		3	1	1	1	1								
	Gb9757	1	1	1	1	1								
		2	0	0	0	0	0	0	0	0	0	0	0	0
		3	1	1	1	1								
G2, Female & Polar Test Item Extract	Gb9758	1	1	1	1	1								
		2	0	0	0	0	0	0	0	0	0	0	0	0
		3	1	1	1	1								
	Gb9759	1	2	2	2	2								
		2	0	0	0	0	0	0	0	0	0	0	0	0
		3	1	1	1	1								
	Gb9760	1	1	1	1	1								
		2	0	0	0	0	0	0	0	0	0	0	0	0
		3	1	1	1	1								
	Gb9761	1	1	1	1	1								
		2	0	0	0	0	0	0	0	0	0	0	0	0
		3	1	1	1	1								
	Gb9762	1	2	2	1	1								
		2	0	0	0	0	0	0	0	0	0	0	0	0
		3	1	1	1	1								
	Gb9763	1	1	1	1	1								
		2	0	0	0	0	0	0	0	0	0	0	0	0
		3	1	1	1	1								
	Gb9764	1	2	2	2	2								
		2	0	0	0	0	0	0	0	0	0	0	0	0
		3	1	1	1	1								
	Gb9765	1	1	1	1	1								
		2	0	0	0	0	0	0	0	0	0	0	0	0
		3	1	1	1	1								
	Gb9766	1	2	2	1	1								
		2	0	0	0	0	0	0	0	0	0	0	0	0
		3	1	1	1	1								
	Gb9767	1	1	1	1	1								
		2	0	0	0	0	0	0	0	0	0	0	0	0
		3	1	1	1	1								

Ery: Erythema; Ede: Oedema; RF: Right flank; 0: No erythema/oedema; hr/hrs: Hour/Hours;
0: No visible change for challenge phase; ant: anterior; post: posterior
Erythema: 1: Very Slight Erythema (barely perceptible); 2: Well Defined Erythema;
Oedema: 1: Very Slight Oedema (barely perceptible); 2: Slight Oedema (edges of area well defined by definite raising)

SKIN REACTIONS SCORING RECORD (Contd…).

Group, Sex & Treatment	Animal No.	Site	Intra-dermal Induction (Day 1)				Topical Induction (Day 8)				Challenge (Day 22)			
			24 (± 2 hrs)		48 (± 2 hrs)		1 hr		24 hrs		24 (± 2 hrs)		48 (± 2 hrs)	
			Ery	Ede	Ery	Ede	Ery	Ede	Ery	Ede	RF ant	RF post	RF ant	RF post
G3, Female & Non-polar Solvent Control	Gb9768	1	1	1	1	1								
		2	0	0	0	0	0	0	0	0	0	0	0	0
		3	1	1	1	1								
	Gb9769	1	1	1	1	1								
		2	0	0	0	0	0	0	0	0	0	0	0	0
		3	1	1	1	1								
	Gb9770	1	1	1	1	1								
		2	0	0	0	0	0	0	0	0	0	0	0	0
		3	1	1	1	1								
	Gb9771	1	1	1	1	1								
		2	0	0	0	0	0	0	0	0	0	0	0	0
		3	1	1	1	1								
	Gb9772	1	1	1	1	1								
		2	0	0	0	0	0	0	0	0	0	0	0	0
		3	1	1	1	1								
G4, Female & Non-polar Test Item Extract	Gb9773	1	1	1	1	1								
		2	0	0	0	0	0	0	0	0	0	0	0	0
		3	1	1	1	1								
	Gb9774	1	1	1	1	1								
		2	0	0	0	0	0	0	0	0	0	0	0	0
		3	1	1	1	1								
	Gb9775	1	1	1	1	1								
		2	0	0	0	0	0	0	0	0	0	0	0	0
		3	1	1	1	1								
	Gb9776	1	2	2	2	2								
		2	0	0	0	0	0	0	0	0	0	0	0	0
		3	1	1	1	1								
	Gb9777	1	1	1	1	1								
		2	0	0	0	0	0	0	0	0	0	0	0	0
		3	1	1	1	1								
	Gb9778	1	1	1	1	1								
		2	0	0	0	0	0	0	0	0	0	0	0	0
		3	1	1	1	1								
	Gb9779	1	2	2	1	1								
		2	0	0	0	0	0	0	0	0	0	0	0	0
		3	1	1	1	1								
	Gb9780	1	1	1	1	1								
		2	0	0	0	0	0	0	0	0	0	0	0	0
		3	1	1	1	1								
	Gb9781	1	1	1	1	1								
		2	0	0	0	0	0	0	0	0	0	0	0	0
		3	1	1	1	1								
	Gb9782	1	2	2	2	2								
		2	0	0	0	0	0	0	0	0	0	0	0	0
		3	1	1	1	1								

Ery: Erythema; Ede: Oedema; RF: Right flank; 0: No erythema/oedema; hr/hrs: Hour/Hours;
0: No visible change for challenge phase; ant: anterior; post: posterior
Erythema: 1: Very Slight Erythema (barely perceptible); 2: Well Defined Erythema;
Oedema: 1: Very Slight Oedema (barely perceptible); 2: Slight Oedema (edges of area well defined by definite raising)

Table S4: Acute ocular irritation test study report

INDIVIDUAL ANIMAL OCULAR REACTIONS SCORING RECORD

Initial Test			Dose: 0.1 mL			Sex: Female			Animal No.: Na8878	
Observation Period	Ocular Lesions									
	Conjunctiva						Iris		Cornea	
	Redness		Chemosis		Discharge				Opacity	
Eyes	LE	RE	LE	RE	LE	RE	LE	RE	LE	RE
1 hr (± 6min)	0	0	0	0	0	0	0	0	0	0
24 (± 2hrs)	0	0	0	0	0	0	0	0	0	0
48 (± 2hrs)	0	0	0	0	0	0	0	0	0	0
72 (± 2hrs)	0	0	0	0	0	0	0	0	0	0
Mean Tissue Scores=(24hr +48hr+72hr)/3	0	0	0	0	0	0	0	0	0	0

Confirmatory Test			Dose: 0.1 mL			Sex: Female			Animal No.: Na8879	
Observation Period	Ocular Lesions									
	Conjunctiva						Iris		Cornea	
	Redness		Chemosis		Discharge				Opacity	
Eyes	LE	RE	LE	RE	LE	RE	LE	RE	LE	RE
1 hr (± 6min)	0	0	0	0	0	0	0	0	0	0
24 (± 2hrs)	0	0	0	0	0	0	0	0	0	0
48 (± 2hrs)	0	0	0	0	0	0	0	0	0	0
72 (± 2hrs)	0	0	0	0	0	0	0	0	0	0
Mean Tissue Scores=(24hr +48hr+72hr)/3	0	0	0	0	0	0	0	0	0	0

Confirmatory Test			Dose: 0.1 mL			Sex: Female			Animal No.: Na8880	
Observation Period	Ocular Lesions									
	Conjunctiva						Iris		Cornea	
	Redness		Chemosis		Discharge				Opacity	
Eyes	LE	RE	LE	RE	LE	RE	LE	RE	LE	RE
1 hr (± 6min)	0	0	0	0	0	0	0	0	0	0
24 (± 2hrs)	0	0	0	0	0	0	0	0	0	0
48 (± 2hrs)	0	0	0	0	0	0	0	0	0	0
72 (± 2hrs)	0	0	0	0	0	0	0	0	0	0
Mean Tissue Scores=(24hr +48hr+72hr)/3	0	0	0	0	0	0	0	0	0	0

min: minutes; hrs: hours; LE: Left Eye (Treated Eye); RE: Right Eye (Untreated)

Conjunctiva - Redness: 0: Vessels Normal

Chemosis: 0: No Swelling

Iris: 0: Normal

Discharge: 0: No Discharge

Cornea - Opacity: 0: No opacity
